# Genetics of phenotypic plasticity and biomass traits in hybrid willows across contrasting environments and years

**DOI:** 10.1093/aob/mcx029

**Published:** 2017-04-25

**Authors:** Sofia Berlin, Henrik R. Hallingbäck, Friderike Beyer, Nils-Erik Nordh, Martin Weih, Ann-Christin Rönnberg-Wästljung

**Affiliations:** 1 Swedish University of Agricultural Sciences, Department of Plant Biology, Uppsala BioCenter, Linnean Centre for Plant Biology, P.O. Box 7080, SE-750 07 Uppsala, Sweden; 2 Swedish University of Agricultural Sciences, Department of Crop Production Ecology, Linnean Centre for Plant Biology, P.O. Box 7043, SE-750 07 Uppsala, Sweden

**Keywords:** SRC willows, biomass, phenology, phenotypic plasticity, QTL mapping, *Salix schwerinii*, *Salix viminalis*, marker-assisted selection

## Abstract

**Background and Aims** Phenotypic plasticity can affect the geographical distribution of taxa and greatly impact the productivity of crops across contrasting and variable environments. The main objectives of this study were to identify genotype–phenotype associations in key biomass and phenology traits and the strength of phenotypic plasticity of these traits in a short-rotation coppice willow population across multiple years and contrasting environments to facilitate marker-assisted selection for these traits.

**Methods** A hybrid *Salix viminalis* × (*S. viminalis* × *Salix schwerinii*) population with 463 individuals was clonally propagated and planted in three common garden experiments comprising one climatic contrast between Sweden and Italy and one water availability contrast in Italy. Several key phenotypic traits were measured and phenotypic plasticity was estimated as the trait value difference between experiments. Quantitative trait locus (QTL) mapping analyses were conducted using a dense linkage map and phenotypic effects of *S. schwerinii* haplotypes derived from detected QTL were assessed.

**Key Results** Across the climatic contrast, clone predictor correlations for biomass traits were low and few common biomass QTL were detected. This indicates that the genetic regulation of biomass traits was sensitive to environmental variation. Biomass QTL were, however, frequently shared across years and across the water availability contrast. Phenology QTL were generally shared between all experiments. Substantial phenotypic plasticity was found among the hybrid offspring, that to a large extent had a genetic origin. Individuals carrying influential *S. schwerinii* haplotypes generally performed well in Sweden but less well in Italy in terms of biomass production.

**Conclusions** The results indicate that specific genetic elements of *S. schwerinii* are more suited to Swedish conditions than to those of Italy. Therefore, selection should preferably be conducted separately for such environments in order to maximize biomass production in admixed *S. viminalis* × *S. schwerinii* populations.

## INTRODUCTION

In heterogeneous environments, including those exposing plants to frequent climate fluctuations, individual plants’ fitness and productivity will depend on their ability to phenotypically adapt to rapidly shifting environments. In such environments, phenotypic plasticity, which is the ability of an organism to alter the expression of a phenotypic trait as a response to changes in environmental conditions (Bradshaw, 1965; Schlichting, 1986) can provide increased environmental tolerance. Therefore, populations composed of highly plastic individuals are expected to be able to inhabit a broad range of environmental conditions or be able to withstand large environmental fluctuations. As a result, phenotypic plasticity is expected to influence the geographical distribution of species (Agrawal, 2001; Sexton *et al.*, 2009).

Phenotypic plasticity of an individual can be depicted by its norm of reaction, which shows the variation of phenotypic traits across different environments, thus displaying the environmental sensitivity of that individual (Schmalhausen, 1949). Individuals of a population can indeed vary in how sensitive they are to environmental changes, which is evident as significant genotype × environment (G × E) interactions, where the extent to which a trait value changes across environments is the phenotypic plasticity (Via and Lande, 1985; Wu, 1998).

As phenotypic plasticity of different traits can influence the performance of individuals across environments, it should be taken into account when conducting selection and breeding for variable climates and environments (Via and Lande, 1985). In order to do this, it is crucial to characterize the level of phenotypic plasticity and its variation among individuals used for breeding and to determine how much of the plasticity has a genetic basis (de Jong and Bijma, 2002). Organisms that can be clonally propagated, e.g. poplars, are excellent study systems as phenotyping can be carried out in clonal replicates grown in contrasting environments (Wu, 1998; Wu and Hinckley, 2001; Fabbrini *et al.*, 2012). Interestingly, clonally replicated poplar populations grown across climatic gradients and contrasting environments have revealed large variation in phenotypic plasticity between clones (Wu and Stettler, 1997, 1998; Rae *et al.*, 2008; Guet *et al.*, 2015; Elferjani *et al.*, 2016). Furthermore, a genetic basis of phenotypic plasticity has also been demonstrated for important biomass traits (Rae *et al.*, 2009; Fabbrini *et al.*, 2012).

Willows in the *Salix* genus are closely related to the poplars and in temperate regions are grown as short-rotation coppice (SRC) primarily for bioenergy production (Kuzovkina *et al.*, 2008). However, improving biomass yield and resistance to pathogens and pests is essential in order to be able to expand willow cultivations to facilitate commercialization. Breeding strategies, especially for perennial crops and trees such as willows, are highly dependent on the level of phenotypic plasticity since selection could be based on high performance across diverse environments aiming for one wide breeding zone or based on high performance in specific environments aiming at different breeding zones.

*Salix viminalis* and *Salix schwerinii* are two closely related willow species (Berlin *et al.*, 2011; Fogelqvist *et al.*, 2015) that are commonly used when breeding for fast-growing and resistant SRC willow cultivars. Hybrid crosses between the two species are easily done experimentally, and many commercial cultivars contain a mix of *S. viminalis* and *S. schwerinii* genetic backgrounds. This is mainly due to a major genetic factor originating from *S. schwerinii* that confers resistance to the leaf rust fungus *Melampsora larici-epitea*, a pathogen that leads to significant yield losses in willow cultivations (Samils *et al.*, 2011). Thus, breeding programmes using both species have successfully developed hybrid cultivars with high biomass productivity and strong resistance to the leaf rust, and that are adapted to the conditions found in Sweden and northern Europe. It is, however, not yet known how hybrid populations are performing in contrasting environments, e.g. Sweden versus southern Europe, and/or in conditions with good water availability versus drought conditions. In particular, it is poorly known whether mixed populations are phenotypically plastic in this regard or whether they harbour any genetic variation for phenotypic plasticity. Addressing such issues becomes increasingly important when adapting breeding populations of long-lived perennial species to future possible climate changes (Alberto *et al.*, 2013).

Keeping this in mind, it should be noted that the identification of genotype–phenotype associations may provide valuable information about genetic regulation of key breeding traits as well as phenotypic plasticity (Nicotra *et al.*, 2010) and may furthermore directly assist breeding for these traits by the use of marker-assisted selection (MAS) (Lande and Thompson, 1990) and genomic selection (Goddard and Hayes, 2007). Genotype–phenotype associations based on the segregation of alleles in bi-parental families can be identified by quantitative trait locus (QTL) mapping analyses using genetic linkage maps. This method can be particularly attractive when investigating largely unexplored traits in non-model organisms with limited genomic resources, as the number of markers required to cover the genome is modest and no *a priori* information about the underlying genes or their function is necessary. QTL mapping analyses using mixed populations have previously been done for biomass (Berlin *et al.*, 2014, Tsarouhas *et al.*, 2002) and phenology traits (Tsarouhas *et al.*, 2003; Ghelardini *et al.*, 2014), as well as for rust resistance (Samils *et al.*, 2011) and drought tolerance (Berlin *et al.*, 2014; Pucholt *et al.*, 2015).

The primary goal of the present study was therefore to study genotype–phenotype associations in key biomass and phenology traits as well as the plasticity of these traits in a hybrid willow pedigree population (called *S*_1_) by QTL mapping analyses. The *S*_1_ population was produced by crossing an *S. viminalis* female (‘78183’) with a *S. schwerinii* (‘79069’) × *S. viminalis* (‘Orm’) hybrid male (cultivar ‘Björn’) (Berlin *et al.*, 2010; Tsarouhas *et al.*, 2002) and the phenotypic assessment of the *S*_1_ progeny was done over multiple years in three common garden experiments located in contrasting environments: one in Sweden and two in Italy (one irrigated and one non-irrigated). The *S. schwerinii* accession 79069 is regarded as a key accession and has been frequently used as an *S. schwerinii* allele donor, usually via the hybrid cultivar ‘Björn’, in willow breeding programmes in northern Europe (Karp *et al.*, 2011). Results from this study are thus essential for identifying markers that are associated with trait variation across a wide range of climates and conditions in order to implement MAS for high-yielding SRC willow cultivars for diverse environments and a changing climate.

## MATERIALS AND METHODS

### Plant material, common garden experiments and phenotypic assessment

In this study, phenotypic and genotypic data from 463 progenies from the *S*_1_ pedigree population were analysed (Tsarouhas *et al.*, 2002; Berlin *et al.*, 2010). The population was produced by crossing the *S. viminalis* female ‘78183’ and the *S. viminalis* × *S. schwerinii* hybrid male ‘Björn’ and is maintained in a plant archive in Pustnäs, South of Uppsala, Sweden (59°49′ N, 17°40′ E). For the study, three different common garden experiments were established (from now on called experiments): one located in Pustnäs, Sweden, and two in Cavallermaggiore, Italy (44°43′ N 7°41′ E). The climatic conditions differed markedly between the two geographical regions. Cavallermaggiore has a higher average annual temperature (12·8 °C) in comparison with Uppsala (5·7 °C) and Cavallermaggiore also had a higher precipitation (746 mm) than Uppsala (551 mm) (www.climate-data.org).

In spring 2008 the experiment in Pustnäs was established, employing a complete randomized block design with six blocks. To establish the experiment, each of the progeny in the *S*_1_ population was clonally propagated by stem cuttings. The cuttings were planted in pots and after 5 weeks of growth in the greenhouse they were planted in the field. One clonal replicate per progeny was planted in every block, so that each progeny was replicated six times (from now on called a ‘clone’). The clonal replicates were randomly positioned in every block. The experiment was manually weeded every year and fertilizer corresponding to 80 kg of N per ha was applied after each cut-down/harvest. The plants were cut down in 2009, 2010, 2011 and 2014 (Table 1, Supplementary Data Table S1).

**Table 1.** Measurements in the different experiments

Trait	Year	Abbreviation	Shoot age/root age		
			Pustnäs	Cavallermaggiore-IRR	Cavallermaggiore-NI
Number of shoots	2009	Nsh09	1/2	–	–
Mean diameter (mm)	2009	MeanD09	1/2	–	–
Summed basal area (mm^2^)	2009	SumBA09	1/2	–	–
Leaf senescence	2010	LS10	2/3	–	–
Number of shoots	2010	Nsh10	2/3	–	–
Fresh weight (kg)	2010	FW10	2/3	–	–
Leaf senescence	2013	LS13	2/6	2/2	2/2
Number of shoots	2013	Nsh13	2/6	2/2	2/2
Mean diameter (mm)	2013	MeanD13	2/6	2/2	2/2
Summed basal area (mm^2^)	2013	SumBA13	2/6	2/2	2/2
Bud burst	2014	BB14	2/6	2/2	2/2
Number of shoots	2014	Nsh14	3/7	3/3	3/3
Mean diameter (mm)	2014	MeanD14	–	3/3	3/3
Summed basal area (mm^2^)	2014	SumBA14	–	3/3	3/3
Fresh weight (kg)	2014	FW14	3/7	–	–

Several biomass traits were assessed on each plant between 2009 and 2014; i.e. the number of shoots (Nsh) in 2009, 2010, 2013 and 2014 and the diameter (D) of each shoot at 105 cm above ground were measured in 2009 and 2013. From these data the mean diameter (MeanD) of each plant was calculated. As a non-destructive measurement of biomass, the summed basal area (SumBA) was calculated for each plant based on the diameter of each shoot (Nordh and Verwijst, 2004). After the harvests in 2010 and 2014, the fresh weight (FW) of each plant was assessed. These fresh weights thus reflect 2 and 3 years of stem growth, respectively. In addition, two phenology traits were measured: leaf senescence (LS) in 2013, according to the scale in Ghelardini *et al.* (2014) and bud burst (BB) in 2014 with the scale used in Weih (2009). Data on LS from 2010 from Ghelardini *et al.* (2014) were added to the analyses for comparison.

In spring 2012, two experiments with contrasting water regimes were established in Cavallermaggiore: one irrigated (Cavallermaggiore-IRR) and one non-irrigated (Cavallermaggiore-NI). To establish the experiments, 12 stem cuttings of 20 cm each were taken from each clone in the Pustnäs experiment in winter 2012. The same experimental layout as in Pustnäs was employed, although the distance between rows was 140 cm (compared with 130 cm in the Pustnäs experiment). One block in the Cavallermaggiore-NI experiment was omitted due to poor survival of the plants. The cuttings were planted on 2 May 2012 in a well-cultivated soil. During the establishment year mechanical weed control was done repeatedly between rows. No fertilizer was applied. Biomass and phenology traits were assessed on each plant in 2013 and 2014, and Nsh was counted and D measured in 2013 and in 2014. We assessed LS and BB in 2013 and 2014, respectively. A description of all measurements in every experiment is presented in Table S1.

### Statistical analyses

To obtain trait predictors for each clone in every experiment, phenotypic data were subjected to variance decomposition by applying mixed linear models with the statistics software ASReml 3·0 (Gilmour *et al.*, 2009). The distributions of the SumBA and FW traits deviated considerably from normal and therefore additional analyses were performed where these traits were transformed by the square root. Nonetheless, because data transformation was observed to have a negligible impact on the results, only the untransformed data analysis results are henceforth treated. The mixed linear model applied was:

(1) $$y_{ijkl}=\mu +b_{i}+c_{j}+{\left(r\times p\right)}_{kl}+e_{ijkl}$$

where *y_ijkl_* is the phenotypic trait value in the *i*th block for the *j*th clone located at row *k* and position *l*. The overall mean is denoted as *μ*, while *b* signifies the fixed block effect, *c* the random clone effect, *r* × *p* the random interaction between rows and positions (spatial term) and *e* the random residual. Random effects were obtained using the best linear unbiased predictor (BLUP) approach (Robinson, 1991) and were assumed to be independent except for the spatial term (*r* × *p*), which was restricted to follow a two-dimensional first-order autoregressive correlation structure (Cullis and Gleeson, 1991) across plant rows and positions. Thus, clone predictors (*y_pr_*) adjusted for block, and spatial environmental effects were obtained from the effect estimates as $y_{pr,j}=\hat{\mu }+{\hat{c}}_{j}$ and used in subsequent analyses.

Because the clone predictors are based on random terms, the broad-sense heritability of the clone predictors ($H_{pr}^{2}$) can be regarded as equivalent to the square of the predictor accuracy (sometimes called *r_TI_*; Henderson, 1984). Thus, following the approach of Wei *et al.* (2003), $H_{pr}^{2}$ for each trait was calculated as:

(2) $$H_{pr}^{2}=1-\frac{\sum_{j=1}^{n}{PE}_{j}^{2}}{n\sigma_{c}^{2}}$$

where (*PE_j_*) is the prediction error of clone *j* supplied by ASReml for each of the *n* tested clones, while $\sigma_{c}^{2}$ is the clone variance estimated by eqn (1).

In order to assess the extent of G × E interactions that truly affect clonal ranking and that are not due to trait scale differences or heterogeneous variances, we estimated Pearson-type clone predictor correlations across experiments (Burdon, 1977). As the clone predictors largely reflect genotypic trait values, such correlations should closely resemble the corresponding genotypic correlations between experiments. Correlation estimates close to zero would thus indicate considerable crossover-type G × E interactions, while estimates close to unity would indicate no or little G × E interaction. Correlations and their 95 % confidence intervals were obtained by using the cor.test function in R (R Core Team, 2013). Plots of clone predictors between contrasting experiments were made with the program JMP^®^ 10 (SAS Institute Inc., 2010).

To get an estimate of the phenotypic plasticity for each clone, a plasticity trait variable was constructed based on standardized (mean = 0, variance = 1) BLUP values by taking the difference between BLUP values in contrasting experiments. Plasticity traits were constructed in this way for BB14, LS13, Nsh13, MeanD13 and SumBA13 between (1) Pustnäs and Cavallermaggiore-IRR, (2) Pustnäs and Cavallermaggiore-NI and (3) Cavallermaggiore-IRR and Cavallermaggiore-NI. A plasticity trait variable for SumBA14 was constructed between Cavallermaggiore-IRR and Cavallermaggiore-NI. In addition, to encompass between-year variation at Pustnäs, plasticity trait variables were constructed for (1) Nsh measured in 2009, 2010, 2013 and 2014 (all possible combinations), (2) SumBA measured in 2009 and 2013, (3) MeanD measured in 2010 and 2014 and (4) FWt measured in 2010 and 2014. In Cavallermaggiore, plasticity trait variables were constructed for Nsh, MeanD and SumBA measured 2013 and 2014.

### QTL mapping analyses

The linkage map developed for the *S*_1_ pedigree population (Tsarouhas *et al.*, 2002; Berlin *et al.*, 2010) was used in QTL mapping analyses. For this study, 41 new markers were added to the map with the JoinMap software (Van Ooijen, 2009). Nineteen markers were positioned on linkage group (LG) XV, one on LG XII, 19 on LG XIX and two on LG XIII. The new markers were developed and genotyped as described in Berlin *et al.* (2010). In total, the linkage map contains 696 markers with a mean distance of 4.4 cM between the markers. QTL mapping was performed using the clone predictors for each trait in each environment and also on the plasticity traits. The program MapQTL 6.0 (Van Ooijen, 2009) with interval mapping and a regression model was used, where the genome was scanned at 1-cM intervals to determine putative QTL associated with the variation of each trait. In order to determine significant QTL, significance threshold values, estimated as the logarithm of the odds ratio (LOD), were determined with a permutation test of 1000 repetitions. A genome-wide threshold for a significant QTL was set at 0·05. One and two LOD confidence intervals for each QTL were estimated using the LOD drop-off method (Lander and Botstein, 1989) based on the LOD value at the peak position of the QTL. The proportion of the clone predictor variation explained by each significant QTL was estimated (PVE %). The difference between maternal alleles was estimated as the absolute effect of:

(3) $$A_{m}=\frac{\left(\mu_{ac}+\mu_{ad}\right)-\left(\mu_{bc}+\mu_{bd}\right)}{2}$$

the differences between paternal alleles were estimated as the absolute effect of:

(4) $$A_{p}=\frac{\left(\mu_{ac}+\mu_{bc}\right)-\left(\mu_{ad}+\mu_{bd}\right)}{2}$$

and the paternal–maternal interaction effect was estimated as the absolute effect of:

(5) $$A_{i}=\frac{\left(\mu_{ac}+\mu_{bd}\right)-\left(\mu_{ad}+\mu_{bc}\right)}{2}$$

where *μ_ac_*, *μ_bc_*, *μ_ad_* and *μ_bd_* are the estimated phenotypic means of the four genotypic classes *ac*, *bc*, *ad* and *bd* obtained from an *ab* ×*cd* cross (Van Ooijen, 2009). For comparison between traits, the effects were calculated as the percentage of the trait mean value. However, for the plasticity QTL the absolute raw effect estimates were retained since the plasticity of traits was based on standardized predictor values. Non-parametric Kruskal–Wallis analyses were performed to verify significant differences between marker genotypic classes close to the peak positions of the QTL. Furthermore, in order to determine phenotypic effects of *S. schwerinii* alleles, haplotypes were constructed for QTL clusters based on phased data from the linkage map and genotypic data from grandparents. Linkage map and QTL positions were depicted using MapChart (Voorrips, 2002).

## RESULTS

### Phenotypic and genetic variation

Across the experiments, plant survival was high; in 2014 survival was 99·9 % in Pustnäs, 96·4 % in Cavallermaggiore-IRR and 93·0 % in Cavallermaggiore-NI. In 2013 the plants grown in Pustnäs (2-year-old shoots, 6-year-old roots) had generally more shoots with lower shoot diameters compared with the plants grown in Cavallermaggiore (2-year-old shoots and roots) (Table 2). This also resulted in a higher shoot biomass in Pustnäs based on the summed basal areas (SumBA13). Independently of treatment, the biomass growth (SumBA13) in Cavallermaggiore was 52 % of the biomass in Pustnäs.

**Table 2.** Arithmetic mean (s.d.) values for each trait measured in Pustnäs, Cavallermaggiore-IRR and Cavallermaggiore-NI

	Location		
Trait	Pustnäs	Cavallermaggiore-IRR	Cavallermaggiore-NI
LS10	1·8 (0·6)	–	–
LS13	1·9 (0·5)	2·9 (0·6)	2·5 (1·0)
BB14	3·5 (0·6)	2·5 (0·8)	2·7 (0·8)
Nsh09	3·7 (1·8)	–	–
Nsh10	4·8 (2·4)	–	–
Nsh13	9·9 (4·7)	3·8 (2·0)	3·7 (2·0)
Nsh14	5·9 (3·2)	4·2 (2·2)	4·1 (2·1)
MeanD09	6·4 (1·5)	–	–
MeanD13	9·0 (1·7)	11·4 (4·1)	11·0 (4·7)
MeanD14	–	16·1 (5·8)	15·8 (6·7)
SumBA09	138·1 (89·4)	–	–
SumBA13	830·8 (479·6)	435·6 (304·1)	435·4 (351·2)
SumBA14	–	996·8 (740·6)	1043·7 (878·1)
FW10	1·092 (0·70)	–	–
FW14	2·800 (1·94)	–	–

Phenotypic variation was observed for all traits and moderate to high broad-sense predictor heritabilities ($H_{\mathrm{pr}}^{2}$ ranging between 0·51 and 0·86) were detected, indicating favourable conditions for detecting QTL (Table 3). Due to the uneven and patchily distributed drought effects, the Cavallermaggiore-NI experiment exhibited a higher percentage of spatial variation (up to 50 %) than the other two experiments (maximum 28 %), resulting in somewhat lower predictor heritability estimates for the NI experiment ($H_{\mathrm{pr}}^{2}$ ranged between 0·51 and 0·82) compared with the other experiments ($H_{\mathrm{pr}}^{2}$ ranged between 0·55 and 0·86) (Table 3).

**Table 3.** Variance component estimates for clone ($\sigma_{c}^{2}$), spatial ($\sigma_{r\times p}^{2}$) and residual ($\sigma_{e}^{2}$) sources of variation plus broad-sense predictor heritabilities ($H_{pr}^{2}$) estimated for each trait assessed in Pustnäs, Cavallermaggiore-IRR and Cavallermaggiore-NI. Component percentages of the total variance are shown in parentheses after the respective component

Trait	$\sigma_{c}^{2}$ (%)	$\sigma_{r\times p}^{2}$ (%)	$\sigma_{e}^{2}$ (%)	$H_{pr}^{2}$
Pustnäs, phenology traits				
LS10	0·145 (41)	0·075 (21)	0·131 (37)	0·86
LS13	0·065 (28)	0·040 (17)	0·129 (55)	0·74
BB14	0·140 (38)	0·051 (14)	0·176 (48)	0·82
Pustnäs, no. of shoots				
Nsh09	0·70 (21)	0·74 (22)	1·94 (57)	0·67
Nsh10	1·15 (21)	0·80 (14)	3·62 (65)	0·65
Nsh13	8·7 (35)	2·9 (12)	12·9 (53)	0·77
Nsh14	3·87 (35)	0·96 (9)	6·28 (56)	0·79
Pustnäs, plant diameters and basal areas				
MeanD09	0·27 (14)	0·38 (19)	1·28 (66)	0·55
MeanD13	0·80 (27)	0·55 (19)	1·60 (54)	0·71
SumBA09	1890 (26)	2021 (28)	3350 (46)	0·74
SumBA13	95 × 10^3^ (38)	35 × 10^3^ (14)	119 × 10^3^ (48)	0·79
Pustnäs, harvest biomass fresh weight				
FW10	0·121 (24)	0·138 (28)	0·242 (48)	0·74
FW14	1·33 (32)	0·79 (19)	2·08 (50)	0·79
Cavallermaggiore-IRR, phenology traits				
LS13	0·100 (28)	0·079 (22)	0·184 (51)	0·67
BB14	0·324 (45)	0·108 (15)	0·280 (39)	0·86
Cavallermaggiore-IRR, biomass traits				
Nsh13	0·99 (25)	0·26 (7)	2·62 (68)	0·67
Nsh14	1·24 (27)	0·22 (5)	3·17 (69)	0·68
MeanD13	5·49 (33)	3·77 (23)	7·32 (44)	0·80
MeanD14	11·6 (35)	5·5 (16)	16·3 (49)	0·79
SumBA13	32·1 × 10^3^ (35)	21·6 × 10^3^ (24)	37·8 × 10^3^ (41)	0·81
SumBA14	212 × 10^3^ (39)	94 × 10^3^ (18)	233 × 10^3^ (43)	0·83
Cavallermaggiore-NI, phenology traits				
LS13	0·078 (11)	0·343 (50)	0·270 (39)	0·51
BB14	0·318 (48)	0·062 (9)	0·289 (43)	0·82
Cavallermaggiore-NI, biomass traits				
Nsh13	0·82 (21)	0·61 (16)	2·41 (63)	0·60
Nsh14	0·97 (22)	0·44 (10)	2·95 (68)	0·59
MeanD13	5·87 (28)	6·84 (33)	8·07 (39)	0·75
MeanD14	13·9 (31)	12·7 (28)	18·4 (41)	0·76
SumBA13	34·5 × 10^3^ (29)	39·0 × 10^3^ (33)	45·5 × 10^3^ (38)	0·75
SumBA14	256 × 10^3^ (34)	178 × 10^3^ (24)	321 × 10^3^ (43)	0·77

Clone predictor correlations across consecutive years were consistently high for all traits (0·72–0·94). In Pustnäs, correlations across multiple years (3–5), including a harvest operation, were also moderate to high (0·53–0·85). The clone predictor correlations between Cavallermaggiore-IRR and Cavallermaggiore-NI ranged from 0·56 (LS) to 0·84 (BB), indicating moderate to low G × E interactions (Table 4). For the BB trait, the correlation between Pustnäs and Cavallermaggiore-IRR was 0·57 and that between Pustnäs and Cavallermaggiore-NI was 0·55. For all other traits, correlations between the Pustnäs and Cavallermaggiore experiments were considerably lower (0·18–0·39) than any correlation estimate across years and irrespective of water availability in Cavallermaggiore (Table 4). These correlation estimates thus indicate considerable crossover-type G × E interactions between Pustnäs and the Cavallermaggiore experiments that cannot be entirely attributed to differences in shoot or root age.

**Table 4.** Clone predictor correlations across pairs of contrasting environments (columns) for the assessed traits (rows) with 95 % confidence intervals in brackets. Pu_2-3_, Pustnäs at root ages 2–3 years (assessed in 2009–2010); Pu_6-7_, Pustnäs at root ages 6–7 years (assessed in 2013–2014). Cavallermaggiore was assessed at root ages 2–3 years only (2013–2014). IRR_2-3_, irrigated Cavallermaggiore experiment; NI_2-3_, non-irrigated Cavallermaggiore experiment

Trait	Pu_2-3_ – IRR_2-3_	Pu_6-7_ – IRR_2-3_	Pu_2-3_ – NI_2-3_	Pu_6-7_ – NI_2-3_	IRR_2-3_ – NI_2-3_
LS	0·30 (0·22–0·38)	0·24 (0·15–0·32)	0·30 (0·21-0·38)	0·27 (0·19–0·35)	0·56 (0·49–0·62)
BB	NI	0·57 (0·51–0·63)	NI	0·55 (0·48–0·61)	0·84 (0·82–0·87)
Nsh	0·18 (0·09–0·26)	0·27 (0·18–0·35)	0·18 (0·10–0·27)	0·23 (0·14–0·31)	0·61 (0·55–0·66)
MeanD	0·34 (0·26–0·42)	0·37 (0·29–0·45)	0·30 (0·22–0·38)	0·39 (0·31–0·46)	0·72 (0·67–0·76)
SumBA	0·25 (0·16–0·33)	0·26 (0·17–0·34)	0·25 (0·16–0·33)	0·26 (0·18–0·34)	0·72 (0·68–0·76)

NI, no information.

**Fig. 1.**
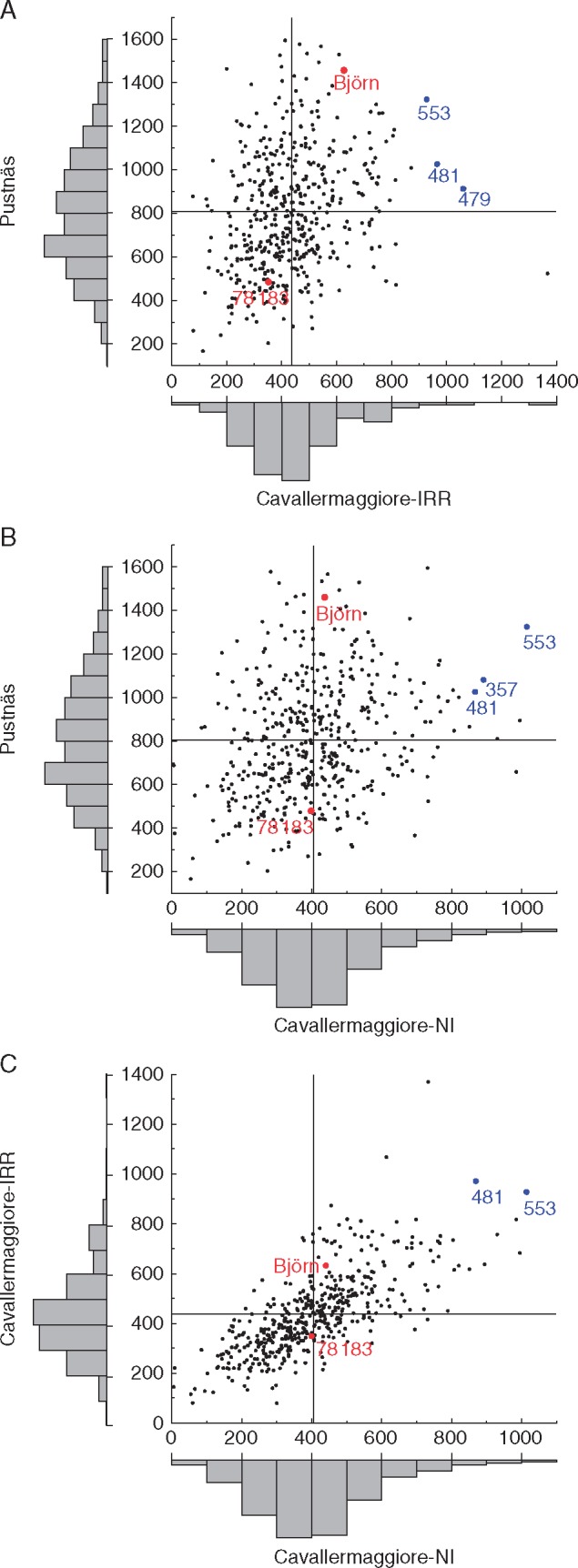
Scatterplots of clone predictors for the trait SumBA13 between contrasting experiments. (A) Pustnäs versus Cavallermaggiore-IRR. (B) Pustnäs versus Cavallermaggiore-NI. (C) Cavallermaggiore-IRR versus Cavallermaggiore-NI. The vertical and horizontal lines in each panel indicate the mean value for each experiment. Plots with distributions of clone predictor values are inserted outside the *x*- and *y*-axes. Red dots indicate male parent (‘Björn’) and female parent (‘78183’) values and blue dots indicate clones with stable and high performance across experiments. The names of key clones are shown beside the respective dots.

The *S*_1_ progeny displayed large variation in phenotypic plasticity for biomass production (based on SumBA13) across the experiments (Fig. 1). Some clones, e.g. 481 and 553, were rather high-producing in all experiments; however, the best-producing clones in Pustnäs were not high-producing in Cavallermaggiore and vice versa.

### QTL are grouped in 18 clusters

**Fig. 2.**
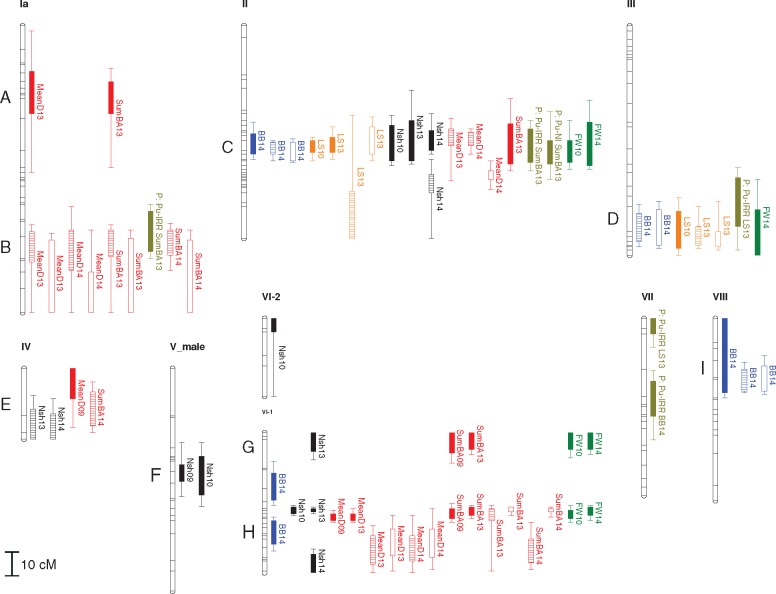

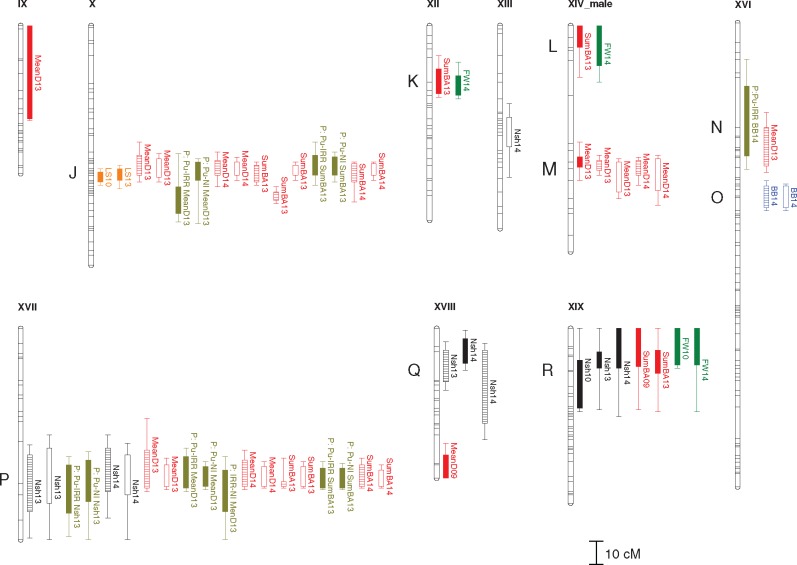
QTL for biomass, phenology and phenotypic plasticity in the experiments in Pustnäs, Cavallermaggiore-IRR and Cavallermaggiore-NI. LOD-1 (bar) and LOD-2 (line) regions from the peak positions of the QTL are indicated. Symbols: filled, Pustnäs (Pu); hatched, Cavallermaggiore-IRR; unfilled, Cavallermaggiore-NI; blue, bud burst; orange, leaf senescence; black, number of shoots; red, biomass traits (SumD, SumBA); dark green, fresh weight; light green, plasticity traits (P). For trait abbreviations see Table 1.

We performed QTL mapping analyses in order to identify genomic regions associated with the regulation of phenology and biomass traits (trait QTL) as well as the plasticity of traits (plasticity QTL). Across all traits and experiments, a total of 133 QTL were identified, of which 126 were clustered into 18 groups with overlapping 2-LOD regions (Table 5, Fig. 2). Three of the clusters, located on LG II (cluster C), X (cluster J) and XVII (cluster P), contained QTL that explained most of the phenotypic variation. In these three clusters, 21 QTL out of 53 explained >10 % of the phenotypic variation (Table 5), thus demonstrating the importance of these regions in the regulation of the investigated traits. Some clusters contained QTL for the same trait measured in different experiments and during different years (clusters C, D, E, H, I, M and Q), indicating that the genetic regulation of these traits was fairly insensitive to year-to-year fluctuations and environmental variation. To analyse the stability of QTL across experiments in greater detail, LOD scores for QTL that were significant in at least one experiment were checked for in the other experiments. This resulted in 14 additional QTL with LOD score >3·0, further supporting the results of common QTL in different experiments (Supplementary Data Table S2). However, some QTL were only found in either Pustnäs or in Cavallermaggiore (Fig. 2, clusters A, B, F, G, J, K, L, O, P and R), which suggests a more complex genetic background with different regulation of the corresponding traits in the different environments.

**Table 5.** Clusters with QTL for biomass and phenology traits in Pustnäs (Pu) and for Cavallermaggiore-IRR (IRR) and Cavallermaggiore-NI (NI)

Cluster	Experiment/ plasticity (P)	Trait	Linkage group	Peak position, cM	LOD	PVE, %	Difference between maternal alleles, % of mean*	Difference between paternal alleles, % of mean*	Interaction effect of alleles, % of mean*
A	Pu	MeanD13	I	43·4	5·0	4·8	3	2	1
A	Pu	SumBA13	I	43·4	5·3	5·2	12	13	2
B	IRR	MeanD13	I	130·3	5·7	5·5	6	6	4
B	NI	MeanD13	I	134·3	6·3	6·0	6	7	5
B	IRR	MeanD14	I	131·3	4·8	4·7	5	7	3
B	NI	MeanD14	I	167·3	5·2	5·0	10	11	8
B	IRR	SumBA13	I	130·3	9·5	9·0	20	15	0
B	NI	SumBA13	I	134·3	5·0	4·8	14	11	7
B	IRR	SumBA14	I	130·3	8·8	8·4	21	17	0
B	NI	SumBA14	I	160·3	4·9	4·7	22	19	9
B	P: Pu-IRR	SumBA13	I	122·4	6·5	6·2	*0·35*	*0·54*	*0·11*
C	Pu	BB14	II	69·8	11·6	10·9	2	6	1
C	IRR	BB14	II	73·7	16·4	15·0	6	14	1
C	NI	BB14	II	73·7	12·1	11·3	5	12	1
C	Pu	LS10	II	71·8	19·6	17·7	4	17	2
C	Pu	LS13	II	69·8	13·0	12·1	3	8	1
C	IRR	LS13	II	120·2	6·4	6·2	1	6	1
C	NI	LS13	II	69·8	6·6	6·3	1	4	1
C	Pu	Nsh10	II	79·3	5·2	5·0	3	6	5
C	Pu	Nsh13	II	74·7	5·3	5·2	6	11	3
C	Pu	Nsh14	II	68·8	8·9	8·5	5	18	1
C	IRR	Nsh14	II	93·5	5·2	5·0	3	10	2
C	IRR	MeanD13	II	64·0	4·9	4·8	2	7	2
C	IRR	MeanD14	II	65·0	7·6	7·2	2	9	3
C	NI	MeanD14	II	89·6	5·8	5·6	1	10	1
C	Pu	SumBA13	II	76·1	4·3	4·1	9	11	2
C	P: Pu-IRR	SumBA13	II	74·7	5·4	5·2	*0·25*	*0·52*	*0·02*
C	P: Pu-NI	SumBA13	II	79·0	4·7	4·6	*0·22*	*0·48*	*0·00*
C	Pu	FW10	II	76·1	4·6	4·4	7	10	5
C	Pu	FW14	II	75·7	4·9	4·8	11	14	0
D	IRR	BB14	III	123·1	6·2	6·0	2	10	1
D	NI	BB14	III	124·7	6·1	5·8	2	9	2
D	Pu	LS10	III	125·7	6·1	5·8	6	8	1
D	IRR	LS13	III	126·7	7·9	7·5	2	4	3
D	NI	LS13	III	128·3	5·1	4·9	1	3	1
D	P: Pu-IRR	LS13	III	108·7	4·7	4·6	*0·49*	*0·32*	*0·35*
D	Pu	FW14	III	124·7	4·7	4·6	17	4	2
E	IRR	Nsh13	IV	33·7	5·2	5·1	3	10	0
E	IRR	Nsh14	IV	34·7	6·1	5·9	4	11	0
E	Pu	MeanD09	IV	0·0	4·6	4·5	3	2	1
E	IRR	SumBA14	IV	27·0	4·7	4·6	2	17	5
F	Pu	Nsh09	V_male	62·0	5·3	5·1	3	9	2
F	Pu	Nsh10	V_male	65·0	5·2	5·0	2	8	2
	Pu	Nsh10	VI-2	0·0	5·4	5·2	7	7	5
G	Pu	Nsh13	VI-1	4·3	5·0	4·9	9	8	1
G	Pu	SumBA09	VI-1	4·3	4·8	4·7	9	8	0
G	Pu	SumBA13	VI-1	4·3	9·1	8·6	11	17	1
G	Pu	FW10	VI-1	4·3	6·6	6·4	10	11	2
G	Pu	FW14	VI-1	4·3	10·2	9·6	13	20	1
	Pu	BB14	VI-1	32·1	5·5	5·3	3	4	1
H	Pu	BB14	VI-1	58·2	5·3	5·1	1	4	1
H	Pu	Nsh10	VI-1	47·0	5·2	5·0	7	2	8
H	Pu	Nsh13	VI-1	46·0	5·4	5·2	9	5	6
H	Pu	Nsh14	VI-1	75·5	4·9	4·7	0	12	5
H	Pu	MeanD09	VI-1	50·8	7·5	7·2	0	4	2
H	Pu	MeanD13	VI-1	49·8	5·3	5·1	1	4	2
H	IRR	MeanD13	VI-1	69·2	6·6	6·3	1	9	3
H	NI	MeanD13	VI-1	65·7	5·6	5·4	1	9	5
H	IRR	MeanD14	VI-1	67·7	7·1	6·8	1	8	6
H	NI	MeanD14	VI-1	64·7	4·7	4·5	1	8	6
H	Pu	SumBA09	VI-1	48·6	5·5	5·3	4	8	16
H	Pu	SumBA13	VI-1	45·6	6·1	5·9	9	12	9
H	IRR	SumBA13	VI-1	49·6	5·0	4·9	8	19	2
H	NI	SumBA13	VI-1	46·0	5·2	5·0	0	13	12
H	IRR	SumBA14	VI-1	70·2	5·2	5·1	10	10	15
H	NI	SumBA14	VI-1	46·0	5·0	4·8	1	15	12
H	Pu	FW10	VI-1	48·6	5·4	5·2	6	7	18
H	Pu	FW14	VI-1	47·0	6·3	6·0	14	15	9
	P: Pu-IRR	LS13	VII	3·0	5·4	5·2	*0·25*	*0·53*	*0·20*
	P: Pu-IRR	BB14	VII	48·5	5·2	5·0	*0·43*	*0·04*	*0·09*
I	IRR	BB14	VIII	39·2	9·2	8·8	10	9	1
I	NI	BB14	VIII	39·2	9·4	8·9	8	10	1
I	Pu	BB14	VIII	20·6	5·8	5·6	2	4	3
	Pu	MeanD13	IX	49·8	5·0	4·9	0	4	0
J	Pu	LS10	X	89·5	10·3	9·8	4	12	1
J	Pu	LS13	X	89·8	6·1	5·9	0	5	1
J	IRR	MeanD13	X	86·9	7·3	7·0	3	9	1
J	NI	MeanD13	X	88·9	9·1	8·6	4	11	1
J	P: Pu-IRR	MeanD13	X	100·3	4·5	4·4	*0·10*	*0·46*	*0·12*
J	P: Pu-NI	MeanD13	X	83·2	5·2	5·1	*0·05*	*0·49*	*0·14*
J	IRR	MeanD14	X	86·9	9·1	8·7	4	10	0
J	NI	MeanD14	X	87·6	11·5	10·8	3	14	1
J	IRR	SumBA13	X	92·5	10·8	10·2	6	23	3
J	IRR	SumBA13	X	102·1	10·6	10·0	18	5	10
J	NI	SumBA13	X	87·6	10·8	10·2	7	25	1
J	P: Pu-IRR	SumBA13	X	83·2	7·4	7·1	*0·23*	*0·56*	*0·32*
J	P: Pu-NI	SumBA13	X	83·2	6·7	6·4	*0·28*	*0·54*	*0·23*
J	IRR	SumBA14	X	92·5	11·2	10·6	7	26	3
J	NI	SumBA14	X	84·4	10·7	10·1	7	29	3
K	Pu	SumBA13	XII	38·7	5·3	5·1	11	5	13
K	Pu	FW14	XII	38·7	4·4	4·3	9	3	15
	NI	Nsh14	XIII	60·3	5·0	4·9	3	5	5
L	Pu	SumBA13	XIV_male	7·0	5·9	5·7	9	10	14
L	Pu	FW14	XIV_male	9·0	5·8	5·6	10	12	15
M	Pu	MeanD13	XIV_male	82·3	4·7	4·6	6	3	3
M	IRR	MeanD13	XIV_male	83·3	8·4	8·0	7	10	5
M	NI	MeanD13	XIV_male	87·9	6·8	6·5	5	10	7
M	IRR	MeanD14	XIV_male	84·3	6·3	6·1	9	9	3
M	NI	MeanD14	XIV_male	85·3	4·6	4·4	9	8	4
N	P: Pu-IRR	BB14	XVI	75·9	4·4	4·3	*0·16*	*0·34*	*0·11*
N	IRR	MeanD13	XVI	80·2	4·8	4·7	6	5	0
O	IRR	BB14	XVI	102·8	7·6	7·3	1	11	2
O	NI	BB14	XVI	102·8	7·9	7·5	1	11	1
P	IRR	Nsh13	XVII	95·2	9·1	8·7	13	10	5
P	NI	Nsh13	XVII	86·9	6·4	6·2	6	9	3
P	P: Pu-IRR	Nsh13	XVII	96·2	10·7	10·1	*0·70*	*0·67*	*0·09*
P	P: Pu-NI	Nsh13	XVII	95·2	7·7	7·4	*0·45*	*0·63*	*0·11*
P	IRR	Nsh14	XVII	86·9	8·3	7·9	11	11	−6
P	NI	Nsh14	XVII	93·2	5·7	5·5	6	7	−5
P	IRR	MeanD13	XVII	88·9	4·4	4·3	7	7	1
P	NI	MeanD13	XVII	91·9	12·5	11·7	5	14	1
P	P: Pu-IRR	MeanD13	XVII	89·9	7·5	7·2	*0·42*	*0·62*	*0·02*
P	P: Pu-NI	MeanD13	XVII	90·9	18·0	16·4	*0·26*	*0·96*	*0·10*
P	P: IRR-NI	MeanD13	XVII	98·2	6·3	6·1	*0·18*	*0·38*	*0·01*
P	IRR	MeanD14	XVII	93·2	5·7	5·5	6	8	2
P	NI	MeanD14	XVII	93·2	14·1	13·1	3	16	1
P	IRR	SumBA13	XVII	91·9	11·9	11·1	0	26	1
P	NI	SumBA13	XVII	89·9	21·3	19·1	4	38	6
P	P: Pu-IRR	SumBA13	XVII	93·2	14·3	13·3	*0·06*	*0·91*	*0·02*
P	P: Pu-NI	SumBA13	XVII	91·9	22·9	20·4	*0·15*	*1·14*	*0·14*
P	IRR	SumBA14	XVII	93·2	10·9	10·2	2	27	4
P	NI	SumBA14	XVII	93·2	17·4	15·9	5	37	−6
Q	IRR	Nsh13	XVIII	21·6	6·7	6·5	12	6	2
Q	Pu	Nsh14	XVIII	13·6	5·2	5·0	12	5	7
Q	IRR	Nsh14	XVIII	23·6	5·1	4·9	11	5	1
	Pu	MeanD09	XVIII	85·2	5·4	5·3	2	2	0
R	Pu	Nsh10	XIX	41·3	4·9	4·7	4	7	5
R	Pu	Nsh13	XIX	19·3	7·2	6·9	3	24	1
R	Pu	Nsh14	XIX	17·3	4·8	4·7	3	21	1
R	Pu	SumBA09	XIX	17·3	4·3	4·2	3	18	5
R	Pu	SumBA13	XIX	18·3	6·0	5·8	3	27	3
R	Pu	FW10	XIX	16·9	6·0	5·8	3	23	5
R	Pu	FW14	XIX	15·9	4·6	4·5	3	26	2

* Raw effects are shown in italics (the plasticity of traits was based on standardized predictor values).

QTL for BB and LS were found in six different clusters (Fig. 2) and clusters C (LG II), D (LG III) and I (LG VIII) harboured QTL for BB and LS assessed in all three experiments, suggesting that these phenology traits are regulated similarly independently of the environment. Furthermore, in cluster C, five of the seven QTL explained >10 % of the phenotypic variation, which demonstrates that cluster C defines a genomic region of particular importance for the phenology traits (Table 5).

Most of the clusters harboured QTL for different biomass traits but only cluster H on LG VI-1 and M on LG XIV contained QTL for MeanD13 and SumBA13 measured in all experiments (Fig. 2), suggesting that some consistency of the genetic regulation of biomass traits across experiments exists even though each QTL explained <10 % of the phenotypic variation. Except for these two clusters, QTL identified for biomass traits in Pustnäs and in Cavallermaggiore mapped in separate clusters. Especially, in clusters J and P several QTL explained between 10 % and 19 % of the phenotypic variation, indicating that these regions were important in regulating biomass production, particularly in Cavallermaggiore (Table 5). With very few exceptions, QTL identified for the two contrasting water treatments in Cavallermaggiore co-located, demonstrating that water availability alone did not impact the genetic regulation of the traits to any great extent (Fig. 2). The overall results rather suggest that the genetic regulation of biomass traits was much more affected by the climatic differences between Pustnäs and Cavallermaggiore than the difference in water availability. Moreover, given the relative consistency of the phenology QTL across experiments, it appears that the genetic regulation of phenology was, in general, less affected by environmental differences than were the biomass traits.

### Plasticity has a genetic origin

A total of 18 plasticity QTL were found between the contrasting experiments and 11 of them originated from the contrast between Pustnäs and Cavallermaggiore-IRR (Fig. 2). Plasticity QTL for the biomass traits (Nsh13, MeanD13 and SumBA13) generally co-located with the corresponding trait QTL in Cavallermaggiore (clusters J and P), whereas the plasticity QTL for phenology traits (BB14 and LS13) most often did not. In particular, MeanD13 and SumBA13 plasticity QTL in cluster P for the contrast between the Pustnäs and Cavallermaggiore-NI explained a considerable part of the phenotypic variation: 16 and 20 %, respectively (Table 5). On the other hand, biomass plasticity QTL very seldom co-located with the corresponding trait QTL detected in Pustnäs (only two cases in cluster C exist). In general this shows that, for the biomass traits, plasticity and trait variation among the progeny grown in Cavallermaggiore were correlated, whereas this was not the case for plasticity and trait variation in Pustnäs.

There was only one significant plasticity QTL for the contrast between Cavallermaggiore-IRR and Cavallermaggiore-NI (Table 5, Fig. 2). This MeanD13 plasticity QTL was located in cluster P close to the corresponding plasticity QTL for the other two contrasts (Pustnäs versus Cavallermaggiore-IRR or Cavallermaggiore-NI), indicating a similarity in the genetic regulation of plasticity independently of contrast.

For the between-year contrasts, eight plasticity QTL were found, of which the majority were for biomass traits in Pustnäs and for longer time intervals, but none of them explained >6 % of the variation (Supplementary Data Table S3).

### Large influence of *S. schwerinii* alleles on phenotypic expression

In order to trace phenotypic effects of *S. schwerinii* alleles and haplotypes, we combined results from the interval mapping analysis, the Kruskal–Wallis analysis and phase information from the linkage map, and, if available, we also used the marker raw data from the genotyping of grandparents (Supplementary Data Table S4). We observed differences in parental allelic effects of significant trait QTL of up to 38 % of the mean value (Table 5, Supplementary Data Table S4) and paternally segregating alleles were associated with differential effects of considerable size more frequently than were maternally segregating alleles. In fact, paternally segregating alleles had differential absolute effects >10 % of the mean values in 54 of the 115 trait QTL, whereas the corresponding number was 22 for maternally segregating alleles. The hybrid origin of the male parent with alleles originating both from *S. viminalis* and from *S. schwerinii* is probably the cause of the frequent occurrence of QTL whose underlying alleles show a high differential effect (Table 6, Table S4). In cluster C, which harboured QTL for both phenology and biomass traits, the effect of *S. schwerinii* alleles was traced by constructing an *S. schwerinii* haplotype for this region. The *S. schwerinii* haplotype gave both an earlier start and earlier leaf senescence independent of experiment. The haplotype also had a positive effect on the number of shoots in all experiments together with a decrease in MeanD in the Cavallermaggiore experiments, ultimately leading to an increase in SumBA13 and FW in Pustnäs only. Paternally segregating alleles associated with large effects were found in cluster P, where specifically SumBA13 and SumBA14 in the Cavallermaggiore experiments showed effects ranging from 26 to 38 % (Table 5, Table S4). These effects are connected to an *S. schwerinii* allele that reduces the biomass growth of the heterozygote genotype (Table 6, Table S4). Similar haplotype effects on SumBA in Cavallermaggiore, although weaker in magnitude (5–29 %), were associated with QTL in cluster J. In cluster R on LG XIX, QTL for different biomass traits measured at Pustnäs entailed effect magnitudes of >20 % in comparison with the mean associated with the replacement of one paternally segregating allele with another (Table 5). Also for plasticity traits, the largest effect magnitudes in general originated from paternally segregating alleles (15 cases out of 18; Table 5). The *S. schwerinii* haplotypes were, for most biomass plasticity QTL (clusters B, J and P), connected to a higher biomass production in Pustnäs relative to production in Cavallermaggiore (Table 6).

**Table 6.** Effects of *S. schwerinii* haplotypes on biomass and phenology traits in selected clusters. Full information is presented in Table S3

Cluster	Trait (Experiment)	Homozygote mean value (maternal genotype)	Heterozygote mean value (paternal genotype)	Phenotypic effect of * S. schwerinii* haplotype
B	SumBA13 (IRR)	468·8	407·7	Decrease
B	SumBA13 (NI)	429·4	383·3	Decrease
B	SumBA13 (P: Pu-IRR)	0·18	0·21	Higher in Pu relative to Ca
B	SumBA14 (IRR)	1098·5	937·9	Decrease
C	BB14 (Pu)	3·30	3·50	Earlier
C	BB14 (IRR)	2·49	2·86	Earlier
C	BB14 (NI)	2·59	2·89	Earlier
C	LS13 (Pu)	2·02	1·89	Earlier
C	LS13 (IRR)	2·88*	3·01*	Earlier
C	LS13 (NI)	2·55	2·45	Earlier
C	Nsh13 (Pu)	9·10	10·09	Increase
C	Nsh14 (Pu)	5·31	6·29	Increase
C	Nsh14 (IRR)	4·34*	3·99*	Increase
C	MeanD13 (IRR)	11·79	10·92	Decrease
C	MeanD14 (IRR)	16·90	15·40	Decrease
C	MeanD14 (NI)	15·79	14·56	Decrease
C	SumBA13 (Pu)	769·4	855·4	Increase
C	SumBA13 (P: Pu-IRR)	−0·24	0·26	Higher in Pu relative to Ca
C	SumBA13 (P: Pu-NI)	−0·19	0·21	Higher in Pu relative to Ca
C	FW10 (Pu)	0·948	1·034	Increase
C	FW14 (Pu)	2·502	2·850	Increase
G	Nsh13 (Pu)	9·22	9·90	Increase
G	SumBA09 (Pu)	128·2	139·1	Increase
G	SumBA13 (Pu)	746·2	873·6	Increase
G	FW10 (Pu)	0·938	1·047	Increase
G	FW14 (Pu)	2·416	2·922	Increase
I	BB14 (Pu)	3·3	3·5	Earlier
J	LS10 (Pu)	1·65*	1·86*	Earlier
J	MeanD14 (NI)	16·35	14·17	Decrease
J	SumBA13 (IRR)	383·9*	492·3*	Decrease
J	SumBA13 (IRR)	474·3	399·6	Decrease
J	SumBA13 (NI)	463·9	354·7	Decrease
J	SumBA13 (P:Pu-IRR)	−0·28	0·30	Higher in Pu relative to Ca
J	SumBA13 (P:Pu-NI)	−0·28	0·28	Higher in Pu relative to Ca
J	SumBA14 (IRR)	880·4*	1165·2*	Decrease
J	SumBA14 (NI)	1127·5	848·5	Decrease
O	BB14 (IRR)	2·8*	2·5*	Earlier
O	BB14 (NI)	2·9*	2·6*	Earlier
P	Nsh14 (IRR)	4·36	4·00	Decrease
P	Nsh14 (NI)	4·22	3·94	Decrease
P	SumBA13 (IRR)	499·3	390·1	Decrease
P	SumBA13 (NI)	485,4	341·9	Decrease
P	SumBA13 (P: Pu-IRR)	−0·50	0·41	Higher in Pu relative to Ca
P	SumBA13 (P: Pu-NI)	−0·61	0·51	Higher in Pu relative to Ca
R	Nsh10 (Pu)	4·68*	5·00*	Decrease
R	Nsh14 (Pu)	5·51*	6·22*	Decrease
R	SumBA13 (Pu)	758·6*	880·8*	Decrease
R	FW14 (Pu)	2·503*	2·895*	Decrease

Pu, Pustnäs; Ca, Cavallermaggiore; IRR, Cavallermaggiore-IRR; NI, Cavallermaggiore-NI.

*Marker in repulsion linkage to other markers specific for *S. schwerinii* in the cluster.

## DISCUSSION

The analyses of variation in key phenotypic traits in SRC willows across multiple environments and years led to the detection of extensive variation in biomass traits and substantial phenotypic plasticity among the offspring in the *S*_1_ hybrid population. Clone predictor correlations within traits but across years were moderate to high for all traits, indicating a fairly consistent genetic regulation over time and that shoot and root ages only have a limited impact. The clone predictor correlations were likewise substantial between the two water contrasts in Cavallermaggiore and, in particular, higher compared with the correlations between the climatic contrasts between Pustnäs and Cavallermaggiore, indicating greater G × E interactions for the climatic contrast compared with the water contrast. The clone predictor correlations thus suggest that the climatic difference influenced the plants to a greater extent than differences in shoot and root ages or the difference in water availability.

We measured phenotypic plasticity for each clone as the difference in standardized trait means across contrasting experiments and between years, and the greater the difference the greater was the phenotypic plasticity. We found different plasticity patterns, as illustrated for the summed basal area in 2013 (SumBA13), where some clones had high values in Cavallermaggiore and low values in Pustnäs while other clones showed the opposite pattern. The occurrence of plasticity QTL for many traits demonstrates that phenotypic plasticity of phenology and biomass traits across the contrasting environments is genetically determined to a substantial degree. As expected from the low clone predictor correlations, most plasticity QTL were found for the two climatic contrasts compared with the water contrast. Interestingly, plasticity biomass QTL often co-located with trait QTL, demonstrating that they are potentially controlled by the same genetic loci. Furthermore, plasticity biomass QTL mostly co-located with the corresponding biomass trait QTL from the Cavallermaggiore experiments and not from Pustnäs, indicating that the variation in plasticity in the population overlaps with the trait variation in Cavallermaggiore, but not in Pustnäs. In contrast, phenology plasticity QTL overlapped only once with the corresponding trait QTL, thus suggesting that the genetic regulation of that plasticity is independent of that of the trait *per se*.

Willow biomass QTL displayed substantial climatic sensitivity as most QTL were detected in either Pustnäs or Cavallermaggiore. The difference between the experiments was also evident by the greater biomass production in Pustnäs compared with Cavallermaggiore. The greater biomass in the Pustnäs experiment in 2013 was influenced by the fact that the plant roots in the Pustnäs experiments were older, and thus better established, than those in Cavallermaggiore when assessed in a given calendar year. Biomass production in Cavallermaggiore was considerably higher in the second year compared with the first year, supporting the idea that establishment as well as root age influence production, which has previously been demonstrated in willows (Nordh, 2005). However, given the considerable G × E interactions observed between Cavallermaggiore and Pustnäs and the environmental specificity of the detected QTL, it cannot be excluded that plants of the *S*_1_ population were better adapted to the local climate in Pustnäs compared with Cavallermaggiore and as a result contributed to a greater accumulated biomass in Pustnäs. The environmental specificity of the genetic background of biomass traits shows how complex these traits are, which means that a large number of genetic loci and multiple pathways are involved in controlling biomass production, and these might vary depending on the environment. Furthermore, given the rather obvious climatic differences between Cavallermaggiore and Pustnäs it is not surprising that different genetic pathways are involved in regulating biomass production. On the other hand, stable QTL were found across years within the same environment for biomass traits, indicating concordant genetic regulation over root and shoot ages, which is furthermore supported by the high clone predictor correlations across years. It should, however, be noted that a few plasticity QTL across years were identified, but they explain very little of the total variation. In taxonomically related poplars, stable biomass QTL were also identified across multiple years (Rae *et al.*, 2009; Muchero *et al.*, 2013).

In contrast to the biomass QTL, most of the phenology QTL were stable across all experiments and years. Phenology QTL in Pustnäs co-located with previously identified QTL for the same traits (Ghelardini *et al.*, 2014), which furthermore supports the stability of QTL across years. Photoperiod (i.e. daylength) alone or in combination with temperature is the main environmental cue regulating both autumn phenology traits (e.g. leaf senescence) and spring phenology traits (e.g. bud burst) (Howe *et al.*, 1996; Keskitalo *et al.*, 2005; Lagercrantz, 2009; Rohde *et al.*, 2011*a*). Previous studies have found that these responses to photoperiod and temperature are under strong genetic control (Bradshaw and Stettler, 1995; Keller *et al.*, 2011; Rohde *et al.*, 2011*b*) and that populations are adapted to the local conditions at the site of growth (Morgenstern, 1996). Local adaptation has been described in several species, and it can be seen as clines in phenology traits (Morgenstern, 1996). Nonetheless, despite phenology QTL consistency, the low and moderate clone predictor correlations between Pustnäs and Cavallermaggiore for leaf senescence and bud burst, respectively, still indicate an influence originating in climatic differences between the two geographical regions. In agreement with this, Fabbrini *et al.* (2012) found considerable G × E interactions for bud set in *Populus nigra* across contrasting environments and concluded that temperature was one of the major causes.

The complex genetic background, particularly for biomass traits, which display extensive environment specificity, leads to major challenges for breeders. A rough trade-off between performance stability across environments and high production (SumBA13) was observed even though a few clones with relatively high biomass production in both experiments in the climatic contrast exist. Thus, it seems that the maximization of biomass production requires separate selection procedures for each environment.

Given the frequent use of a specific *S. schwerinii* genetic donor cultivar (‘79069’) in the breeding of willows for biomass, we were interested to know what phenotypic effects haplotypes originating from that *S. schwerinii* parent actually had on the measured traits. Also, here we found a complex pattern, as phenotypic effects of *S. schwerinii* haplotypes differed among QTL clusters and experiments. However, *S. schwerinii* haplotypes derived from QTL always conferred an early bud burst and early leaf senescence independent of experiment. Moreover, *S. schwerinii* haplotypes were often associated with increased biomass production in Pustnäs, but mostly with decreased biomass production in Cavallermaggiore. In terms of biomass production, this implies that several genetic key elements of *S. schwerinii* confer poorer plant adaptation to the climate in northern Italy in comparison with the corresponding *S. viminalis* elements, but such a pattern was not observed in Sweden. This is further supported by the observation that the *S. schwerinii* haplotypes for plasticity of biomass traits always conferred a lower biomass production in Cavallermaggiore in relation to that of Pustnäs. Moreover, the biomass plasticity QTL co-located frequently with the regular trait QTL whose *S. schwerinii* haplotypes *per se* decreased biomass production in Cavallermaggiore. Since *S. schwerinii* has a north-eastern distribution in Asia (Skvortsov, 1999) and does not occur naturally in Sweden or Italy, the climate is probably more similar to Swedish conditions compared with northern Italian conditions. Our results thus suggest that individuals comprising a heavy genetic *S. schwerinii* component could readily be used when breeding for a more northerly climate but would likely not be the best suited for a southern one.

The rationale behind comparing environments with contrasting climate is the desire to develop clonal cultivars that contain the valuable rust resistance factors originating from *S. schwerinii* but that nonetheless are suitable also for southern European conditions. The stability of some of the QTL indicates the possibility of using these QTL in MAS even though for biomass traits the effect of the *S. schwerinii* allele appears to vary depending on environment. Given the patterns and characteristics of the QTL detected and the considerable G × E interactions (low clone predictor correlations) for the climatic contrast, it seems reasonable to separate the breeding material into two different breeding populations aimed at the different environments (Namkoong *et al.*, 1988). In MAS the same allele could be selected for or against depending on the breeding objective and, because we identified QTL that could be used specifically in separate environments for both biomass and phenology traits, we conclude that divergent breeding in divided populations could even be facilitated by MAS.

Further studies are needed to be able to select markers within QTL for use in MAS, but these are the first important findings to identify genomic regions for willow biomass traits across contrasting environments in Europe.

## SUPPLEMENTARY DATA

Supplementary data are available online at https://academic.oup.com/aob and consist of the following. Table S1: management and measurements in the field experiments. Table S2: overview of QTL across environments and environmental contrasts (plasticity). Table S3: summary of QTL analyses of across-year plasticity traits for all experiments. Table S4: grandparental genotypes and Kruskal–Wallis test for markers in peak positions of selected clusters.

## Supplementary Material

Supplementary Data
